# Correlation of vaccine‐elicited antibody levels and neutralizing activities against SARS‐CoV‐2 and its variants

**DOI:** 10.1002/ctm2.644

**Published:** 2021-12-19

**Authors:** Jinbiao Liu, Brittany H Bodnar, Nigam H Padhiar, Adil I Khan, Fengzhen Meng, Sami Saribas, Peng Wang, Xu Wang, Elizabeth McCluskey, Sahil Shah, Huaqing Zhao, Jin Jun Luo, Wen‐Hui Hu, Wen‐Zhe Ho

**Affiliations:** ^1^ Department of Pathology and Laboratory Medicine, Lewis Katz School of Medicine, Temple University Philadelphia Pennsylvania USA; ^2^ Center for Metabolic Disease Research, Lewis Katz School of Medicine, Temple University Philadelphia Pennsylvania USA; ^3^ Department of Biomedical Education and Data Science, Lewis Katz School of Medicine, Temple University Philadelphia Pennsylvania USA; ^4^ Department of Neurology Lewis Katz School of Medicine Temple University Philadelphia Pennsylvania USA

Dear Editor,

The COVID‐19 vaccines (Pfizer‐BNT162b2 and Moderna‐mRNA‐1273) can elicit an effective immune response against severe acute respiratory syndrome coronavirus 2 (SARS‐CoV‐2) infection.^1^, ^2^ However, titers of elicited serum antibody to spike protein of the virus differ among vaccinated individuals and decline after vaccination.^3^, ^4^ Additionally, the ability of the vaccines to protect against newly emerged variants needs to be further elucidated. Therefore, it is important to understand the correlation between levels of vaccination‐induced antibody and neutralizing activity against SARS‐CoV‐2, including the variants.

Previous authors have investigated the subject of vaccine efficacy against SARS‐CoV‐2 variants using both clinical and in‐vitro models. For example, a study by Bernal et al. comparing the B.1.617.2 and B.1.1.7 variants using clinical data noted only modest differences in BNT162b2 and ChAdOx1 vaccine's effectiveness.^5^ Abu‐Raddad et al. used a similar test‐negative case‐control design and found that the BNT162b2 vaccine's effectiveness was reduced against B.1.351, but noted that protection against severe disease was still robust.^6^ An in‐vitro study using BNT162b2‐elicited serum by Liu et al. reported a roughly equivalent neutralization of B.1.1.7 and P.1 variants when compared to USA‐WA1/2020 and slightly lower (but still robust) neutralization for B.1.351.^7^ Another in‐vitro study by Chen et al. reported reductions in neutralizing activity against B.1.1.7 and B.1.351 variants when examining geometric mean titers (GMTs) using BNT162b2 derived serum.^8^ Data from Stamatatos et al. examining the neutralizing ability of sera from 15 donors vaccinated with either Pfizer/BioNTech BNT162b2 or Moderna mRNA‐1273 demonstrated that the two mRNA vaccines have reduced potency against divergent variants, specifically B.1.351.^9^ In summary, these authors have noted decreased vaccine efficacy against the B.1.351 variant. However, few of these studies included a large cohort of Moderna mRNA‐1273 vaccinated donors, and fewer still attempted to compare the neutralizing ability of Moderna mRNA‐1273 elicited serum to BNT162b2 elicited serum when looking at both the Wuhan‐1 reference isolate and its variants. These prior studies also did not attempt to show any correlation between serum Immunoglobulin G (IgG) levels and neutralizing ability.

We thus examined levels of vaccine‐elicited serum antibody and neutralizing activities against pseudoviruses bearing spike proteins from the original Wuhan‐1 reference isolate (wild type, WT) and the variants (D614G, UK‐B.1.1.7, UK‐B.1.525 and SA‐B.1.351) (Table S1). We obtained sera samples from 30 mRNA‐BNT162b2 (Pfizer) vaccinated subjects (22–68 days after 2nd dose) and 19 mRNA‐1237 (Moderna) vaccinated subjects (24–49 days after 2nd dose) (Table S2). This study was approved by Temple University Institutional Review Board (IRB; IRB #28021) and the informed consent forms were signed by all study subjects.

We measured the serum titers of specific IgG antibodies to SARS‐CoV‐2 spike S1 by an enzyme‐linked immunosorbent assay and demonstrated that all subjects vaccinated with either Pfizer or Moderna vaccine had detectable levels of serum IgG. The Pfizer group IgG titers ranged from 1.05 × 10^4^ to 1.68 × 10^5^ ng/ml, and the Moderna group IgG titers ranged from 2.01 × 10^4^ to 1.70 × 10^5^ ng/ml (Figure 1A). Given that the distribution of the IgG titers was left‐skewed (Figure 1B), we reported the GMTs, with the geometric standard deviation factor (GSDF) and the 95% CI of the GMT. For the Pfizer group, the GMT was 6.12 × 10^4^ ng/ml (GSDF = 2.24, 95% confidence interval [CI] = 4.53 × 10^4^–8.27 × 10^4^ ng/ml). For the Moderna group, the GMT was 9.24 × 10^4^ ng/ml (GSDF = 1.90, 95% CI = 6.78 × 10^4^–1.26 × 10^4^ ng/ml). The Wilcoxon rank‐sum test *p*‐value comparing both groups’ IgG titers was 0.0906, and thus the difference in post‐vaccination titers between Pfizer and Moderna recipients was not statistically significant (*p* > 0.05).

**FIGURE 1:**
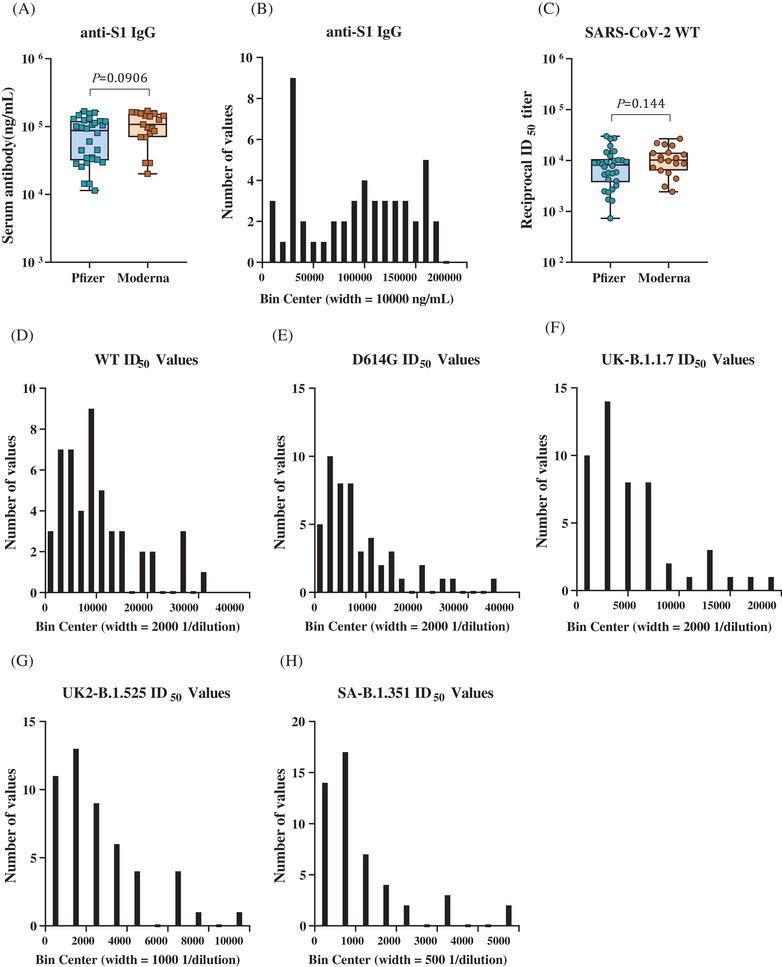
Distribution of specific anti‐severe acute respiratory syndrome coronavirus 2 (anti‐SARS‐CoV‐2) S1 Immunoglobulin G (IgG) in serum from vaccine recipients and distribution of ID_50_ values for each variant. (A) Shown is specific anti‐SARS‐CoV‐2 S1 IgG in sera collected from Pfizer (*N* = 30) and Moderna (*N* = 19) vaccinated subjects approximately 3 weeks to 2 months after the second dose of vaccination. A Wilcoxon rank‐sum test was used for the *p*‐value calculation. (B) Shown is the distribution of all of the serum IgG titers from 49 donors. (C) Shown is 50% pseudovirus neutralization titer (50% inhibitory dilution, ID_50_) against recombinant vesicular stomatitis virus‐based SARS‐CoV‐2 pseudovirus bearing the Wuhan‐1 (wild type, WT) spike protein in sera collected from Pfizer and Moderna vaccinated subjects. Box plots indicate the median and interquartile range (IQR); the whiskers represent 1.5 times the IQR. The unit for reciprocal ID_50_ is 1/dilution. A Wilcoxon rank‐sum test was used for the *p*‐value calculation. (D–H) Shown is the distribution of neutralizing ID_50_ values for each variant. Bin width was individually determined for each graph to avoid over‐smoothing of data

We then performed a recombinant vesicular stomatitis virus‐based SARS‐CoV‐2 neutralization assay to determine the serum neutralization titer (50% inhibitory dilution, ID_50_, units are 1/dilution) of all vaccinated subjects. The neutralizing infectivity of the pseudoviruses was evaluated in sera at dilutions ranging from 1:50 to 1:36 450. We showed that sera from all vaccinated subjects had neutralizing activity and that there was no statistical difference (*p* = .144) in serum neutralizing activity (ID_50_) against SARS‐CoV‐2 WT between Pfizer and Moderna (Figure 1C). For the Pfizer group, the WT ID_50_ ranged from 732 to 3.00 × 10^4^, and for the Moderna group, the WT ID_50_ ranged from 2.43 × 10^3^ to 2.67 × 10^4^ (Figure 1C). As the ID_50_ values are left‐skewed for all variants (Figure 1D–H), we have also reported the GMT of the ID_50_ values for each group with the GSDF and 95% CI of the GMT. The Pfizer group WT ID_50_ had a GMT of 6.74 × 10^3^ (GSDF = 2.42, 95% CI = 4.84 × 10^3^–9.38 × 10^3^), and the Moderna group ID_50_ had a GMT of 9.67 × 10^3^ (GSDF = 1.92, 95% CI = 7.07 × 10^3^–1.32 × 10^4^). Therefore, based on the large GSDF, there is substantial variation in the neutralizing ID_50_ values for both groups of vaccine recipients for WT SARS‐CoV‐2. It is worth noting that the level of neutralizing antibodies that confers immunity in‐vivo against SARS‐CoV‐2 is not well known, and so it is possible that even those donors with low ID_50_ values (and therefore, comparatively poorer neutralizing ability) would still have sufficient antibody titers to negate or diminish viral infection and its associated sequelae.

Although sera from all vaccinated subjects could neutralize the pseudoviruses bearing spike proteins of variants, neutralizing titers were lower when compared to SARS‐CoV‐2 WT spike protein. In Pfizer‐vaccinated sera, there was a significant decrease of GMTs for D614G (–1.45‐fold, 95% CI = 1.21–1.74), B.1.1.7 (–2.20‐fold, 95% CI = 1.81–2.78), B.1.525 (–4.06‐fold, 95% CI = 3.33–4.96), and B.1.351 (–10.5‐fold, 95% CI = 8.56–12.8), respectively (Figure 2A). In Moderna‐vaccinated sera, although there was little difference between GMT of SARS‐CoV‐2 WT and that of D614G (–1.06‐fold, 95% CI = .761–1.48), there was a significant reduction of GMTs for B.1.1.7 (–1.59‐fold, 95% CI = 1.22–2.08), B.1.525 (–3.37‐fold, 95% CI = 2.57–4.39), and B.1.351 (–8.04‐fold, 95%CI = 6.36–10.2), respectively (Figure 2B). Among the variants studied, B.1.351 appeared to be the most resistant to the neutralization by sera from either Pfizer (reduction of 10.5‐fold) or Moderna (reduction of 8.04‐fold) groups. This finding is consistent with and supported by recent reports.^10^ Despite an overall decline in neutralizing titers (GMTs) against the variants, sera at low dilution (1:50) could neutralize 99% of both SARS‐CoV‐2 WT pseudovirus and the variants (D614G, B.1.1.7, B.1.525 and B.1.351) (Figures S1 and S2). The linear regression analysis showed a significant and positive correlation between serum IgG levels and neutralizing activities (ID_50_) against SARS‐CoV‐2 WT (goodness of fit [*R* ^2^]^ ^= 0.282, *p *< 0.001) or variants: D614G (*R* ^2 ^= 0.326, *p *< 0.001), B.1.1.7 (*R* ^2^ = 0.286, *p *< 0.001), B.1.525 (*R* ^2 ^= 0.290, *p *< 0.001) and B.1.351 (*R* ^2 ^= 0.230, *p *< 0.001), respectively (Figure 2C).

**FIGURE 2:**
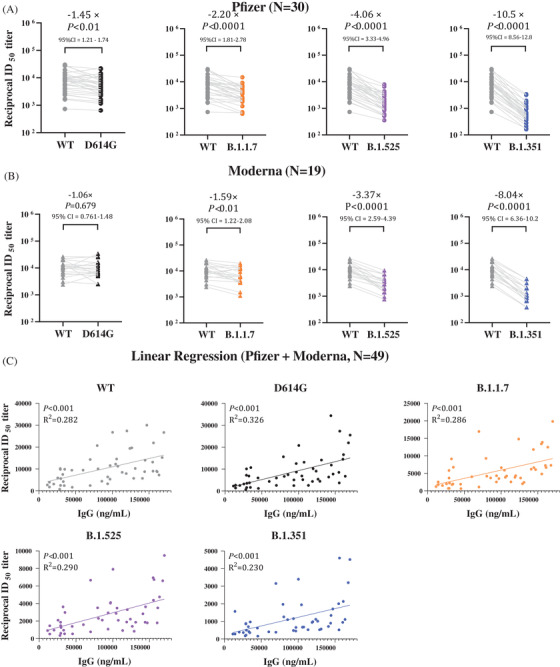
Neutralization of severe acute respiratory syndrome coronavirus 2 (SARS‐CoV‐2) pseudoviruses in sera and its correlation with vaccine‐elicited IgG levels. Sera obtained from either Pfizer or Moderna vaccinated subjects were collected three weeks to two months after the second dose vaccine. Neutralization was measured in an assay with recombinant vesicular stomatitis virus (rVSV)‐based pseudovirus bearing spike proteins of SARS‐CoV‐2 WT or the full‐set variants. (A, B) Shown is the reciprocal neutralizing titers at a 50% inhibitory dilution (ID_50_). The lines connect the WT and variant neutralizing titers in matched samples. Fold changes in the reciprocal geometric mean ID_50_ in vaccinated sera against the D614G, B.1.1.7, B.1.525 and B.1.351 variants, as compared with WT, are shown above the *p‐*value. The dots in Panel A indicate the sera ID_50_ titers of Pfizer vaccinated subjects; the triangles in Panel B indicate the sera ID_50_ titers of Moderna vaccinated subjects. The grey, black, orange, purple and blue symbols represent the ID_50_ titer of the WT, D614G, B.1.1.7, B.1.525 and B.1.351 variants, respectively. The numbers over the dot of each group are the geometric mean titers (GMTs). (C) Shown is the correlation of the neutralizing titers ID_50_ (abscissa) and anti‐SARS‐CoV‐2 spike S1 IgG levels (ordinate) of sera from vaccinated subjects. (Pfizer, *N* = 30; Moderna, *N* = 19). In Panel A and Panel B, the Wilcoxon matched‐pairs signed‐rank test was used for two‐group analysis. In Panel C, linear regression analysis was performed using GraphPad Prism 9.1.1. software. Pearson's correlation coefficients were calculated. Simple linear regression (solid line) is shown. *R* ^2^ = goodness of fit. *p‐*values less than 0.05 are statistically significant

In conclusion, we demonstrated that all study participants vaccinated with either Pfizer or Moderna vaccine were able to produce effective antibodies against spike proteins of both SARS‐CoV‐2 WT and the variants (Table S3). There was a several‐fold reduction in GMTs of ID_50_ against the variants (UK‐B.1.1.7, UK‐B.1.525 and SA‐B.1.351) in sera, as compared to those against SARS‐CoV‐2 WT. However, sera at low dilutions were equally effective in neutralizing both SARS‐CoV‐2 and the variants. Importantly, we demonstrated that among all vaccinated subjects, there was an overall positive correlation between serum IgG levels and ID_50_ titers for not only SARS‐CoV‐2 WT but also the variants (Figure 2C). This finding suggests that the level of IgG titer may be a correlate of immunity. Therefore, it is necessary to longitudinally monitor specific serum IgG levels for evaluating the protective efficacy of vaccines against SARS‐CoV‐2 and its new variants.

Limitations of the study include the small sample size, the lack of live SARS‐CoV‐2 neutralization assays, and the fact that the timing of sampling after the second dose of mRNA vaccine was not well controlled (ranging from 22 to 68 days)

## CONFLICT OF INTEREST

The authors declare that they have no conflict of interest.

## ADDITIONAL CONTRIBUTIONS

We thank all the voluntary vaccinated subjects for providing clinical samples and the members of the Neurology Clinic in Temple Hospital for collecting blood samples. We also thank Guangxiang Luo, PhD (University of Alabama), for providing Hela/ACE2‐11 cells. None of these contributors received any compensation for their help in carrying out the study.

## Supporting information

Supporting Information
