# Supplementary material for: Correlation of vaccine‐elicited antibody levels and neutralizing activities against SARS‐CoV‐2 and its variants
Source: Clin Transl Med. 2021 Dec 19;11(12):e644. doi: 10.1002/ctm2.644 (PMC8684769; doi:10.1002/ctm2.644)
Supplement: Supplementary file 1 — Supporting Information [file CTM2-11-e644-s001.docx]

**Supporting information**

**Correlation of vaccine-elicited antibody levels and neutralizing activities against SARS-CoV-2 and its variants**

Jinbiao Liu, Ph.D.^1^, Brittany H Bodnar, M.S.^1,2^, Nigam H Padhiar, B.S.^1^, Adil I Khan, Ph.D.^1^, Fengzhen Meng, M.S.^1^, Sami Saribas, Ph.D.^1,2^, Peng Wang, Ph.D.^1^, Xu Wang, M.S.^1^, Elizabeth McCluskey, B.S.^1^, Sahil Shah. B.S.^1,2^, Huaqing Zhao, Ph.D.^3^ Jin Jun Luo, M.D., Ph.D.^4^*, Wen-Hui Hu, M.D., Ph.D.^1,2^*, and Wen-Zhe Ho, M.D., M.P.H.^1^*

Table of Contents

[Materials and Methods 2](#_Toc85750368)

[Ethical Approval 2](#_Toc85750369)

[Subjects Enrolled and Human Samples 2](#_Toc85750370)

[Recombinant VSV-based Pseudovirus Neutralization 2](#_Toc85750371)

[SARS-CoV-2 Spike Human IgG ELISA 2](#_Toc85750372)

[Statistical Analysis 3](#_Toc85750373)

[Figure S1 4](#_Toc85750374)

[Figure S2 6](#_Toc85750375)

[Table S1 7](#_Toc85750376)

[Table S2 7](#_Toc85750377)

[Table S3 8](#_Toc85750378)

[Contributions 9](#_Toc85750379)

[Acknowledgements 9](#_Toc85750380)

[Conflict of Interest Disclosures: None reported. 9](#_Toc85750381)

[References 9](#_Toc85750382)

# Materials and Methods

## Ethical Approval

The study was approved by the Institutional Review Board of Temple University (IRB number: #28021). All the study subjects signed written informed consent.

## Subjects Enrolled and Human Samples

All the enrolled subjects were given two-doses of either Pfizer or Moderna vaccine on a prime-boost schedule. Peripheral blood samples were collected three weeks to two months after the second dose of vaccine. The sera neutralizing activity against wild type and variants of SARS-CoV-2 was measured.

## Recombinant VSV-based Pseudovirus Neutralization

A codon-optimized spike protein of the original Wuhan isolate (WT) was cloned into pCAGGS vector. To enhance the packaging efficiency of pseudovirus, the 18 aa at C-terminal of spike was deleted. All the mutations were introduced into the spike gene using one or two steps of NEBuilder HiFi cloning. The plasmids were sequenced to ensure that only the intended mutations were present. To generate recombinant vesicular stomatitis virus (rVSV)-*firefly*-luciferase pseudovirus bearing spike of SARS-CoV-2 S-WT and variants, BHK-21/WI-2 cells (Kerafast, EH1011) were transfected with the spike expression plasmid and subsequently infected with rVSV-*firefly*-luciferase (Kerafast, EH1020-PM) as previously described^1,2^. The pseudovirus-containing culture supernatant was harvested and centrifuged at 500 g for 5 min to remove cell debris. The cell-free supernatant was then filtered through a 0.45 μm polyethersulfone membrane and stored at -150°C in 100 μL aliquots for future use.

For the neutralization assay, Hela/ACE2-11 cells (Gift from Dr. Guangxiang Luo, University of Alabama) were seeded in 96-well plates in culture medium and allowed to reach approximately 85% confluence before use in the assay (24 h later). Sera were 3-fold serially diluted in medium (dilutions ranged from 1:50 to 1:36,450) and incubated with VSV based-pseudovirus at 37 °C for 30 minutes. The virus-sera mix was subsequently used to infect Hela/ACE2-11 cells for 36 h at 37 °C after which cells were washed with PBS and lysed with Passive Lysis Buffer (Promega). Firefly luciferase activity (relative luminescence unit; RLU) in lysates was measured using the Luciferase Assay System (Promega) with EnVision Multimode Plate Reader (PerkinElmer). The obtained RLU was normalized to those derived from cells infected with pseudovirus only. The half-maximal inhibitory dilution for serum (ID_50_) was defined as 1/dilution, and was determined using four-parameter logistic curve (GraphPad Prism Version 9.1.1).

## SARS-CoV-2 Spike Human IgG ELISA

The SARS-CoV-2 Spike S1 human IgG from vaccinated human sera was quantified with ELISA following the protocol of the manufacturer (Biolegend, Cat# 447807). Briefly, human sera were diluted at 1:5000 and 1:10000 with assay buffer, then added to SARS-CoV-2 S1 pre-coated wells and incubated at room temperature for 2 h, followed by incubation with biotinylated SARS-CoV-2 S1 detection antibody for 1 h and Avidin-HRP for 30 min. Peroxidase substrate solution (TMB) and 1M H_2_SO_4_ stop solution were used and the absorbance (OD 450 nm and 570 nm) was read by a microplate reader (Spectra Max i3, Molecular Devices, Sunnyvale, CA, USA). Lyophilized SARS-CoV-2 Spike S1 human IgG was 2-fold serially diluted starting from 15 ng/mL and used as standard for accurate quantitation of SARS-CoV-2 Spike S1 human IgG.

## Statistical Analysis

GraphPad Prism software (Version 9.1.1) and Stata (Version 14) were used for statistical analysis. Histograms were used to examine the distribution of the serum and ID_50_ values (bin widths were selected to minimize over-smoothing of data, based on automatically selected values from GraphPad Prism^TM^ software). The antilog of the mean of log(WT/variants) was used to calculate fold change for each variant. Wilcoxon signed rank test was used for paired two-group analysis. Wilcoxon rank-sum test was used for two independent group comparisons. Pearson’s correlation coefficients were calculated for the regression between serum IgG values and ID_50_ values for each variant . *P* values less than 0.05 were statistically significant. The geometric mean standard deviation factor, a multiplicative & dimensionless measure of variation, was calculated for each geometric mean, as well as the 95% confidence intervals. Detailed descriptive statistics can be found in **Table S3**.

# Figure S1


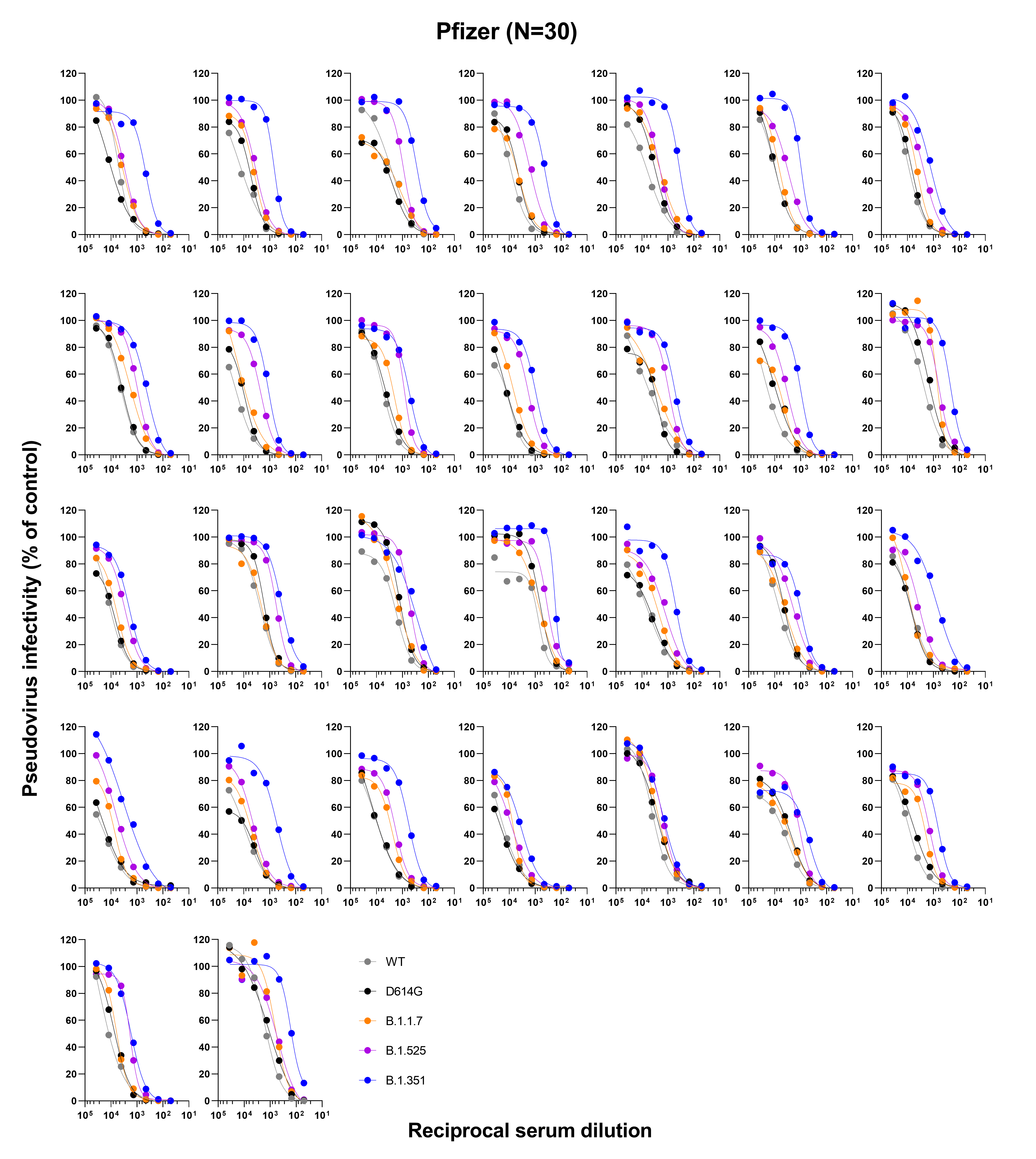


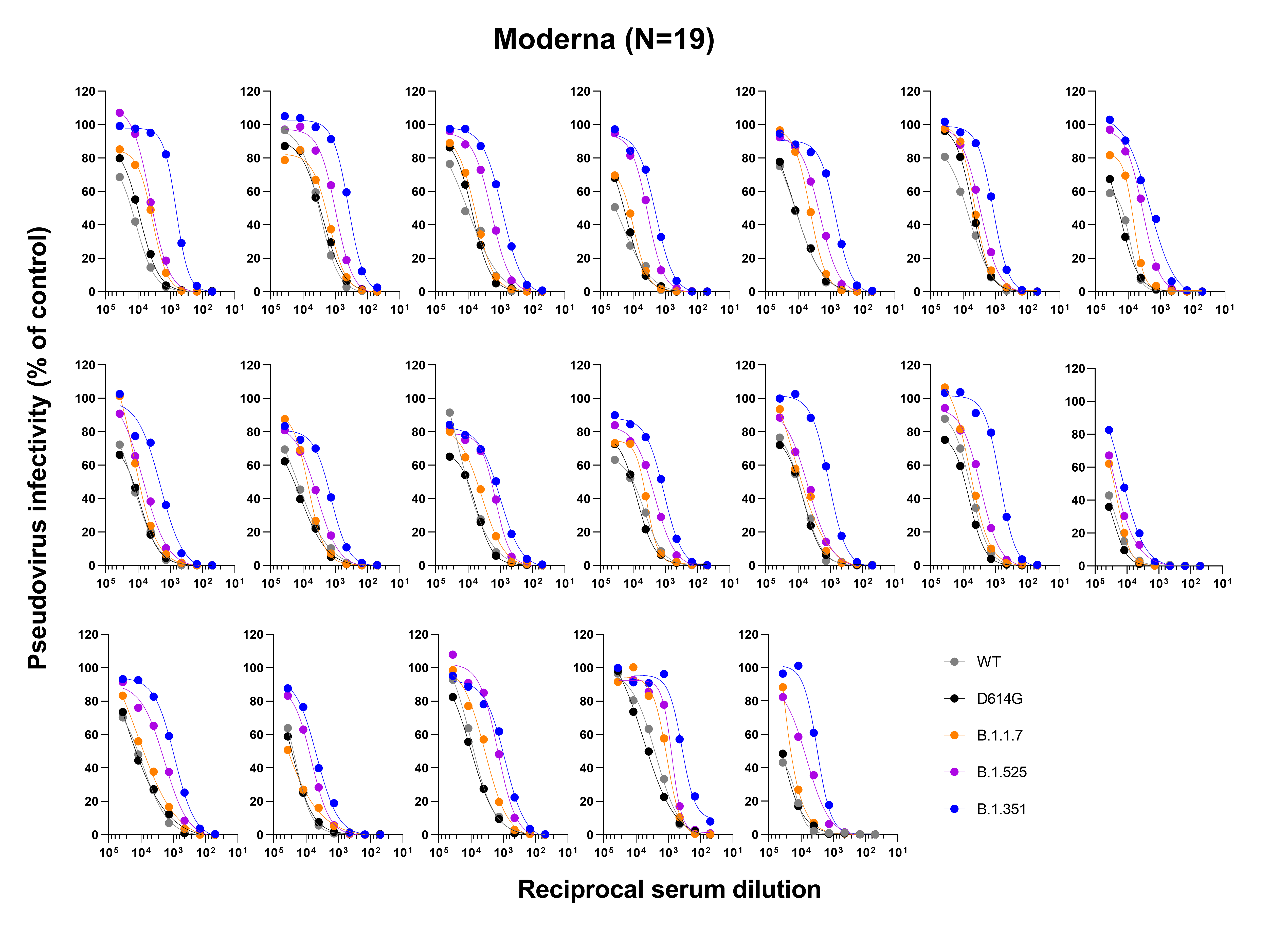


**Figure S1. Neutralization curves of human sera in the VSV-based pseudovirus neutralization assay.**

Sera were collected from Pfizer (N=30) and Moderna (N=19) vaccinated subjects approximately 3 weeks to 2 months after second dose of vaccination. Neutralization was measured against recombinant VSV-based pseudovirus bearing the full-length spike protein of SARS-CoV-2 WT, D614G, B.1.1.7, B.1.525, or B.1.351 variant. Each graph represents an individual participant, as indicated. Each data point is an average from duplicate wells. The obtained RLU were normalized to those derived from cells infected with pseudovirus only (control), and subsequently analyzed using four-parameter logistic curve (GraphPad Prism Version 9.1.1).

# Figure S2


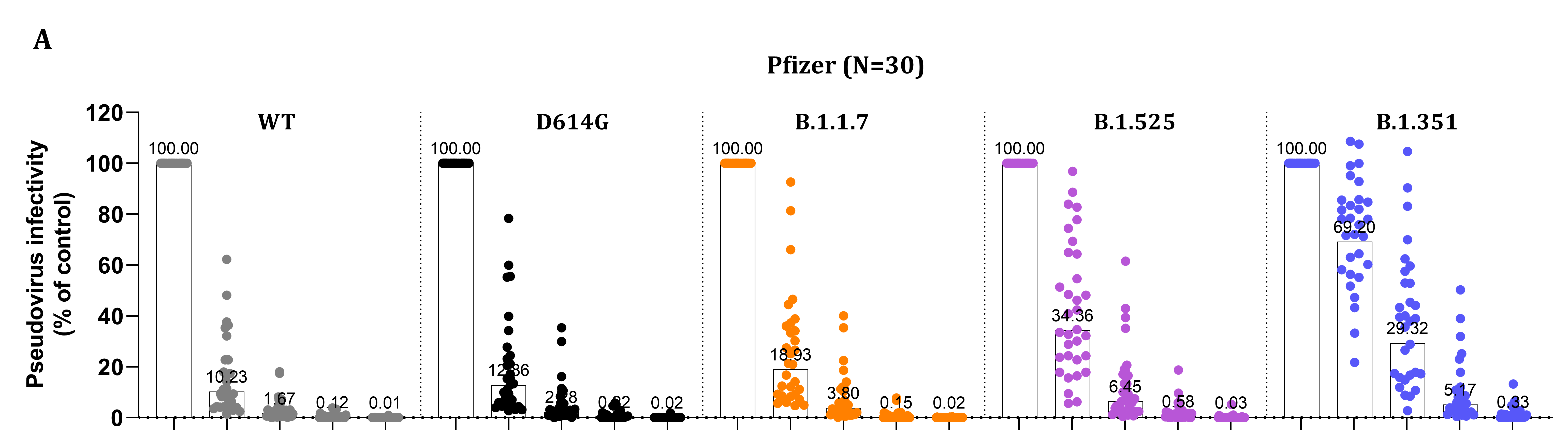

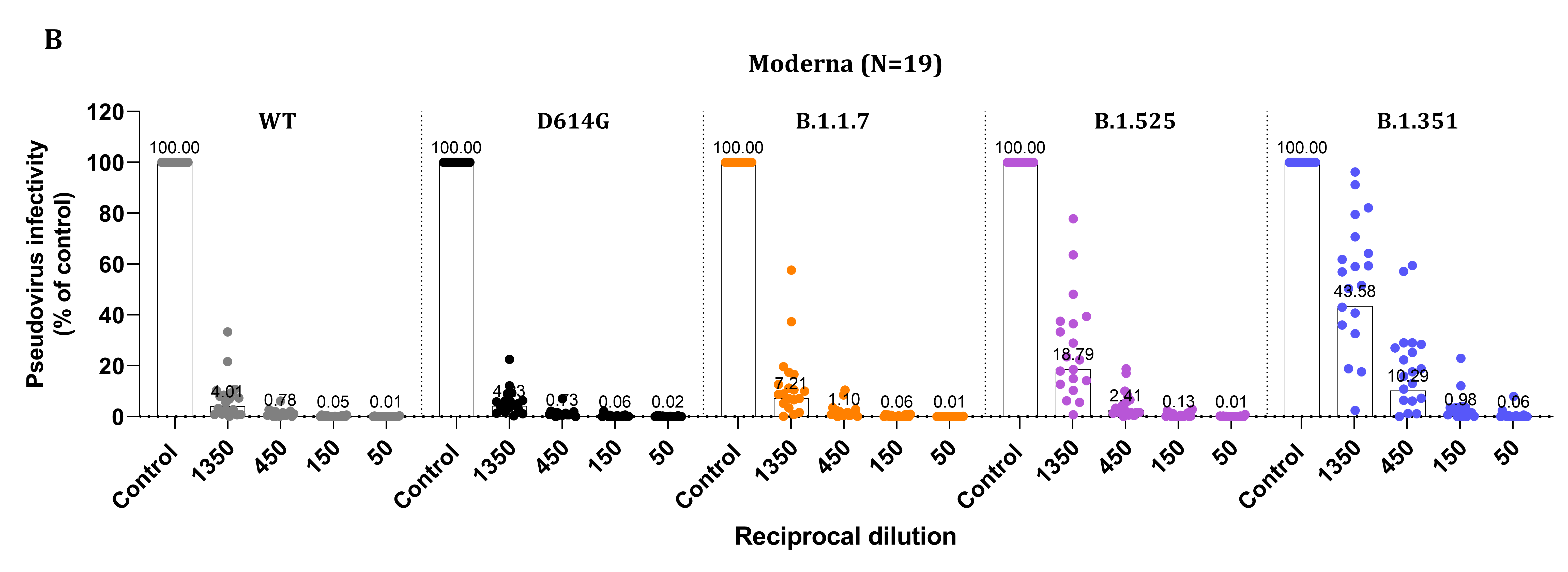


**Figure S2. Neutralization activity of diluted sera against SARS-CoV-2 WT and its variants.**

Sera were collected from Pfizer (N=30) and Moderna (N=19) vaccinated subjects, and 3-fold serially diluted in medium starting with a 1:50 dilution. Neutralization was measured against recombinant VSV-based pseudovirus bearing the full-length spike protein of SARS-CoV-2 WT, D614G, B.1.1.7, B.1.525, or B.1.351 variant. Firefly luciferase activity (relative luminescence unit; RLU) in lysates was measured using the Luciferase Assay System (Promega) with EnVision Multimode Plate Reader (PerkinElmer). The obtained RLU was normalized to those derived from cells infected with pseudovirus only (control). Each dot represents an individual participant. The numbers over the dot of each group are the geometric mean of the relative infectivity.

# Table S1

**Spike mutations in SARS-CoV-2 variants**

| **SARS-CoV-2** | **Amino Acid Changes in Spike** |
| --- | --- |
| **Wild Type (WT)**  (Wuhan-1 reference strain) |  |
| **B.1** | D614G |
| **B.1.1.7**  (20I/501Y.V1, VOC 202012/01) | ∆HV69-70-∆Y144-N501Y-A570D-D614G-P681H-T716I-S982A-D1118H |
| **B.1.351**  (20H/501Y.V2) | L18F-D80A-D215G-∆LAL242-244-R246I- K417N-E484K-N501Y-D614G-A701V |
| **B.1.525**  (20C) | Q52R-∆HV69-70-∆Y144-E484K-D614G-Q677H-F888L |

# Table S2

**Characteristics of study subjects**

| **Characteristics** | **Pfizer vaccinee** | **Moderna vaccinee** |
| --- | --- | --- |
| **No. of subjects** | 30 | 19 |
| **Age (median, range)** | 36.0 (21.0-73.0) | 39.0 (20.0-65.0) |
| **Sex** |  |  |
| Male (%) | 11 (36.7) | 7 (36.8) |
| Female (%) | 19 (63.3) | 12 (63.2) |
| **Days post 2nd Dose (Median day, range)** | 30.5 (22-68) | 35.0 (24.0-49.0) |
| **Race** | White: 16  Black or African American: 2  Asian: 12 | White: 11  Black or African American: 1  Asian: 7 |

# Table S3

**Descriptive Statistics for Pfizer IgG titers (ng/mL) and ID_50_ results (1/dilution)**

|  | **Serum IgG Titers** | **WT ID_50_** | **D614G ID_50_** | **B.1.1.7 ID_50_** | **B.1.351 ID_50_** | **B.1.525 ID_50_** |
| --- | --- | --- | --- | --- | --- | --- |
| **Range** | **1.14E+04-1.68E+05** | **732-3.00E+04** | **650-2.20E+04** | **628-1.48E+04** | **162-3.40E+03** | **357-7.92E+03** |
| **Median**  (95% CI) | **8.71E+04**  (3.44E+04-1.13E+05) | **8.17E+03**  (5.31E+03-9.86E+03) | **4.38E+03**  (3.17E+03-6.85E+03) | **3.04E+03**  (2.03E+03-4.35E+03) | **547**  (452-944) | **1.79E+03**  (1.08E+03-2.30E+03) |
| **IQR** | **3.18E+04-1.21E+05** | **3.75E+03-1.07E+04** | **2.53E+03-9.34E+03** | **1.94E+03-5.94E+03** | **396-1.02E+03** | **917-3.06E+03** |
| **Geometric Mean**  (GSDF, 95% CI) | **6.12E+04**  (2.24,  4.53E+04-8.27E+04) | **6.74E+03**  (2.42, 4.84E+03-9.38E+03) | **4.65E+03**  (2.40, 3.35E+03-6.45E+03) | **3.06E+03**  (2.16, 2.29E+03-4.08E+03) | **644**  (2.17, 482-859) | **1.66E+03**  (2.18, 1.24E+03-2.22E+03) |

**Descriptive Statistics for Moderna IgG titers (ng/mL) and ID_50_ results (1/dilution)**

|  | **Serum IgG Titers** | **WT ID_50_** | **D614G ID_50_** | **B.1.1.7 ID_50_** | **B.1.351 ID_50_** | **B.1.525 ID_50_** | |
| --- | --- | --- | --- | --- | --- | --- | --- |
| **Range** | **2.01E+04-1.70E+05** | **2.43E+03-2.67E+04** | **2.53E+03-3.44E+04** | **1.12E+03-1.98E+04** | **381-4.60E+03** | **770-9.49E+03** |  |
| **Median**  (95% CI) | **1.08E+05**  (6.98E+04-1.55E+05) | **1.02E+04**  (6.35E+03-1.43E+04) | **8.49E+03**  (5.92E+03-1.56E+04) | **6.19E+03**  (3.69E+03-1.25E+04) | **1.10E+03**  (710-2.01E+03) | **2.94E+03**  (1.86E+03-4.76E+03) | |
| **IQR** | **6.98E+04-1.55E+05** | **6.35E+03-2.67E+04** | **5.92E+03-1.56E+04** | **3.69E+03-1.25E+04** | **710-2.01E+03** | **1.86E+03-4.76E+03** | |
| **Geometric Mean**  (GSDF, 95% CI) | **9.24E+04**  (1.90, 6.78E+04-1.26E+05) | **9.67E+03**  (1.92, 7.07E+03-1.32E+04) | **9.10E+03**  (2.05, 6.44E+03-1.29E+04) | **6.07E+03**  (2.18, 4.17E+03-8.85E+03) | **1.20E+03**  (2.05, 852-1.70E+03) | **2.87E+03**  (2.00, 2.06E+03-4.01E+03) | |

# Contributions

Conceptualization, J.B.L., W.Z.H., W.H.H, and J.J.L.; methodology, J.B.L, B.H.B, F.Z.M., S.Sa., P.W., E.M., S.Sh., N.H.P., and W.H.H.; sera samples, A.I.K., X.W., and J.J.L.; statistical analysis, N.H.P, H.Z., J.B.L., and W.Z.H.; writing-original draft preparation, J.B.L., and W.Z.H.; writing-review and editing, N.H.P., W.Z.H., W.H.H, and J.J.L. All authors have read and agreed to the published version of the manuscript.

# Acknowledgements

We thank all the voluntary vaccinated subjects for providing clinical samples; members of Neurology Clinic in Temple Hospital for collecting blood samples; Guangxiang Luo, Ph.D. (University of Alabama), for providing Hela/ACE2-11 cells. None of these contributors received any compensation for their help in carrying out the study.

# Conflict of Interest Disclosures: None reported.

# References

1. Liu J, Bodnar BH, Meng F, et al. Epigallocatechin Gallate from Green Tea Effectively Blocks Infection of SARS-CoV-2 and New Variants by Inhibiting Spike Binding to ACE2 Receptor. BioRxiv 2021:2021.03.17.435637.

2. Whitt MA. Generation of VSV pseudotypes using recombinant ΔG-VSV for studies on virus entry, identification of entry inhibitors, and immune responses to vaccines. J Virol Methods 2010;169:365-74.
